# Choroidal evaluation in patients under alpha-lytic therapy

**DOI:** 10.1007/s00417-020-04907-1

**Published:** 2020-09-02

**Authors:** Maddalena De Bernardo, Vincenzo Altieri, Alessia Coppola, Marco Gioia, Nicola Rosa

**Affiliations:** Department of Medicine, Surgery and Dentistry “Scuola Medica Salernitana”, University of Salerno, via S. Allende, 84081 Baronissi, Salerno Italy

**Keywords:** Choroidal thickness, IFIS, Cataract surgery, OCT, Alpha-lytic therapy

## Abstract

**Purpose:**

To detect any choroidal thickness (CT) change in patients after alpha-lytic drugs withdrawal that could help in the cataract surgery timing decision.

**Methods:**

Twenty-five eyes of 25 patients (mean age: 76 ± 7 years) under alpha-lytic therapy, and 25 eyes of 25 control subjects (CS) (mean age: 75 ± 7 years) without alpha-lytic therapy, both scheduled for cataract surgery in the fellow eye, were included in this observational, prospective, non-randomized study. All patients underwent EDI-OCT during the first preoperative visit and approximately 1 month (range 28–31 days) after alpha-lytic withdrawal. In the CS group, the OCT during preoperative visit and approximately 1 month after (range 28–31 days) the first examination was performed. Data normality with Kolmogorov-Smirnov test was checked and statistical evaluation with the Wilcoxon-signed rank test was performed.

**Results:**

The mean subfoveal CT was 224 ± 79.7 μm during therapy and 217 ± 70.4 μm after withdrawal; 1.5 mm nasally from the fovea CT was 198 ± 83.8 μm and 194 ± 82.8 μm, respectively; and 1.5 mm temporally from the fovea CT was 217 ± 55.9 μm and 205 ± 54.4 μm, respectively. A statistically significant reduction (*p* < 0.05) in all the 3 measured CT points was found. In the CS no significant changes were detected (*p* > 0.05).

**Conclusion:**

No severe floppy iris syndrome was detected at the time of surgery. In these patients, CT decrease could be an important sign for cataract surgery timing decision.

## Introduction


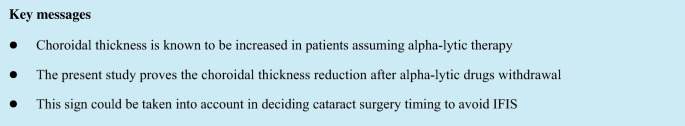
Cataract represents the leading cause of blindness worldwide [1]. Today, due to the phacoemulsification techniques and the improvements in intraocular lens (IOL) calculations, cataract surgery is considered one of the most successful procedures in ophthalmology [2].

An adequate pupillary dilatation and the iris stability are among the reasons that make this surgery successful.

Sympathetic system, through noradrenaline release, induces radial iris muscle contraction and subsequent pupil dilation (mydriasis). Unfortunately, several drugs, such as selective α1 adrenergic receptor antagonists (ARA) inhibitors, can complicate cataract surgery, inducing miosis with iris stroma surging and billowing. Even with normal intraocular fluid flows and despite well-constructed wounds, iris could prolapse through surgical incisions. This phenomenon has been described with the name of intraoperative floppy iris syndrome (IFIS) [3].

Ophthalmologists must be aware of IFIS because it is associated with high rates of intraoperative complications, such as iris prolapse, capsulorhexis tear, iris trauma, anterior chamber hemorrhage, zonula dehiscence, posterior capsule rupture, and vitreous loss, as well as postoperative complications, including intraocular pressure elevation and cystoid macular edema [4, 5].

To try to avoid the IFIS onset, the withdrawal of selective α1ARA inhibitors before surgery has been suggested. Unfortunately, such discontinuation is not always effective and no objective preoperative signs, which help to forecast the success in avoiding IFIS have been described.

As the whole uvea could be involved in the drug-induced modifications, the purpose of this study was to check if such a withdrawal could produce choroidal thickness (CT) changes.

## Methods

In the present observational, prospective, non-randomized study, patients scheduled for cataract surgery in the Eye Department of the University of Salerno, taking alpha-lytic therapy, and a control group were included. The study was consistent with the tenets of the Declaration of Helsinki; institutional ethics committee approval and informed consent were obtained from all participants. Patients affected by corneal leukomas, diabetes, maculopathy, central serous chorioretinopathy, and optic neuropathy were excluded. Twenty-five eyes of 25 consecutive patients with a mean age of 76 ± 7 years, ranging from 58 to 85 years, were evaluated, of whom twenty-four male patients with a previous diagnosis of Benign Prostatic Hypertrophy (BPH), whose urinary symptoms were controlled by alpha-lytic therapy (twelve patients took Tamsulosin, seven Alfuzosin and others five Silodosin) and one female with Arterial Hypertension (AH), controlled by Doxazosin. For comparison, a control group of 25 patients with a mean age of 75 ± 7 years, ranging from 61 to 86 years that were not on alpha-lytic therapy, was utilized.

In both groups, because of dense lens opacities that could cause fictitious and inaccurate results, or possible artifacts induced by surgery, the fellow eyes were examined, whereas the operated eyes were excluded. The day of preoperative visit, all patients underwent complete eye examination, including uncorrected and best spectacle corrected visual acuity, anterior segment evaluation, intraocular pressure (IOP), fundus examination, axial length (AL) measurement with an IOLMaster (5.4.4.0006; Carl Zeiss Meditec AG), and OCT examination with Heidelberg Spectralis OCT using EDI in 840 nm (Software version 5.3; Spectralis SD-OCT; Heidelberg Engineering, Heidelberg, Germany). Scans were acquired using a single horizontal line scan, with a scanning angle of 308 and consisted of 36 frames per B-scan using the average real time mode (ART). Only scans through the fovea and images with a high signal-to-noise ratio (minimum of 20 dB) were included and used for the CT evaluation. In order to reduce the IFIS risk, after consultation with urologist or cardiologist, patients were asked to discontinue alpha-lytic therapy and not to change their lifestyles or other therapies. The day of the surgery, performed approximately 1 month later (range 28–31 days), the OCT examination was repeated. Utilizing the OCT software, the measurements were obtained by an expert examiner, not aware of the patients’ distribution. A line connecting the RPE outer edge and the scleral inner edge was drawn, being perpendicular to the line tangential to the foveal contour. The measurements were performed at subfoveal level, at 1.5 mm nasally and temporally to the fovea. All data were analyzed with SPSS Software (IBM SPSS Statistics version 25). The normal data distribution was assessed with Kolmogorov-Smirnov test. Sample size was determined by maximizing the statistical power. The analysis was performed by using G*Power3.1 software [6]. A difference between two dependent means (matched pairs) Wilcoxon rank test was computed. Input data were the following: α was set at 0.05, 1-β was set at 0.80, and effect size was set as medium at around 0.6. Results were the following: non-centrality parameter δ = 2.932, critical *t* = 2.069, DF = 22.873, actual power = 0.80, and total sample size = 25.

## Results

**Fig. 1:**
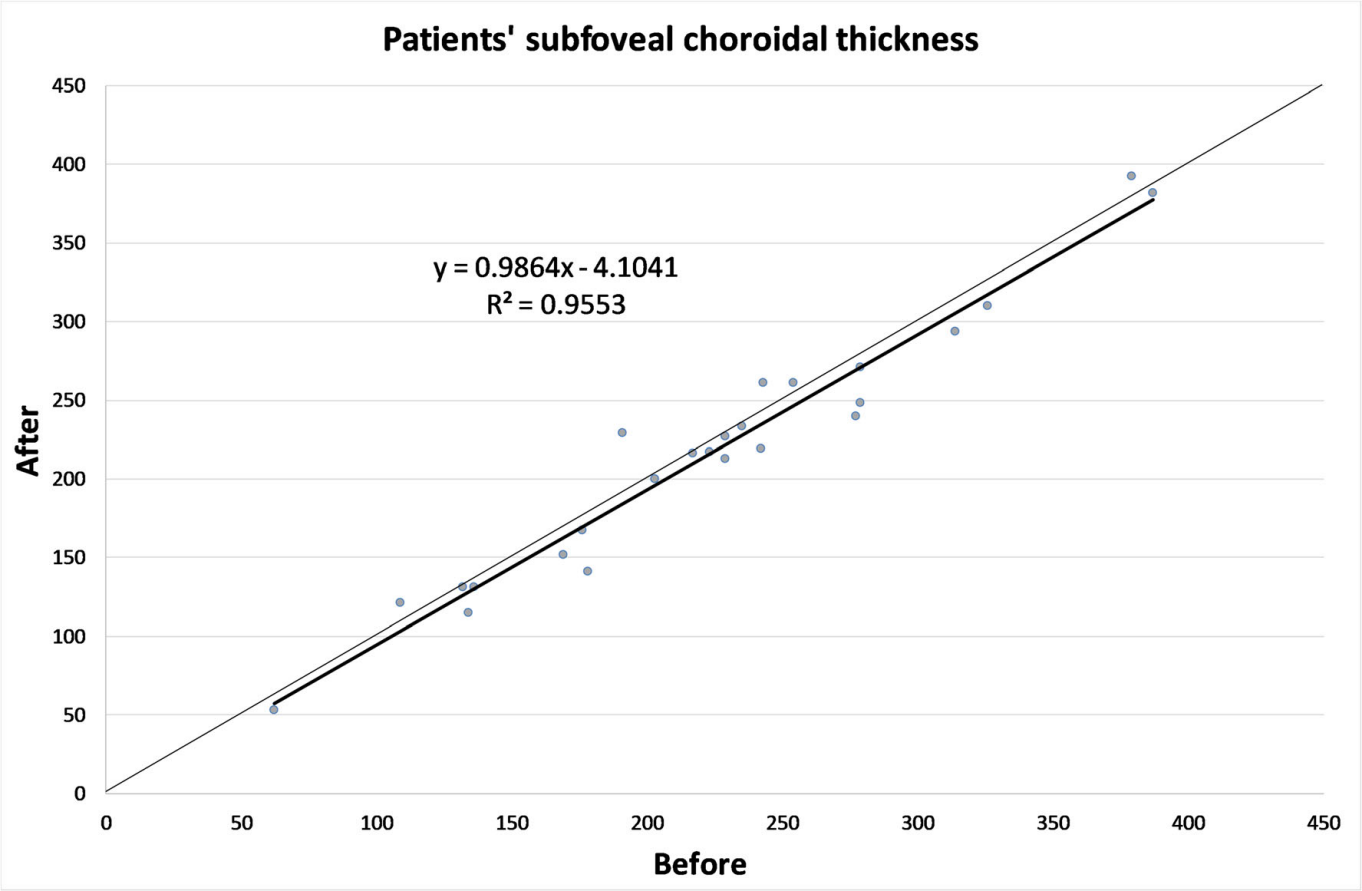
Patients’ choroidal thickness at the subfoveal level before and after alpha-lytic therapy withdrawal

**Fig. 2:**
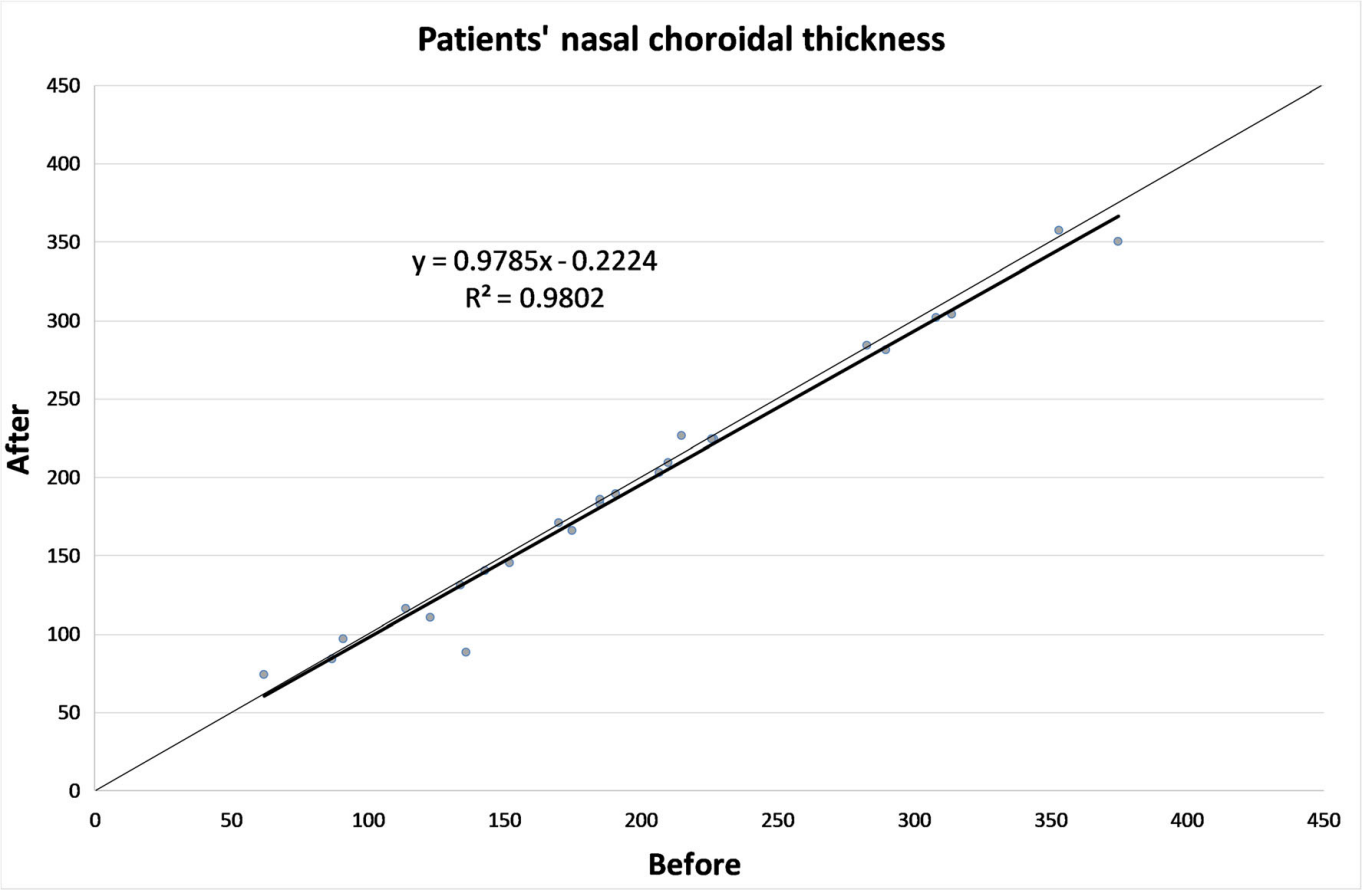
Patients’ choroidal thickness at 1.5 mm nasally from the fovea before and after alpha-lytic therapy withdrawal

**Fig. 3:**
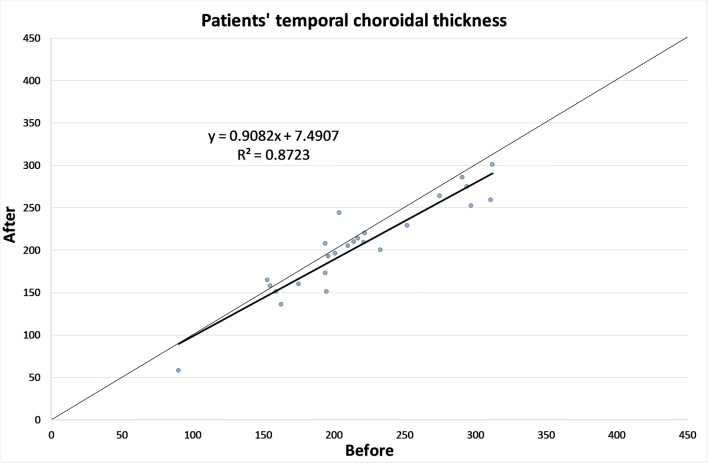
Patients’ choroidal thickness at 1.5 mm temporally from the fovea before and after alpha-lytic therapy withdrawal

**Fig. 4:**
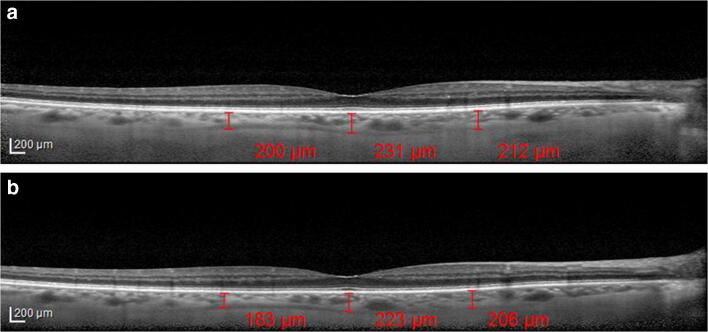
**a** Choroidal thickness evaluated at the sub-foveal level, at 1.5 mm nasally and temporally from the fovea in a patient under alpha-lytic treatment. **b** Choroidal thickness evaluated at the subfoveal level, at 1.5 mm nasally and temporally from the fovea in the same patient 1 month after alpha-lytic therapy withdrawal

**Fig. 5:**
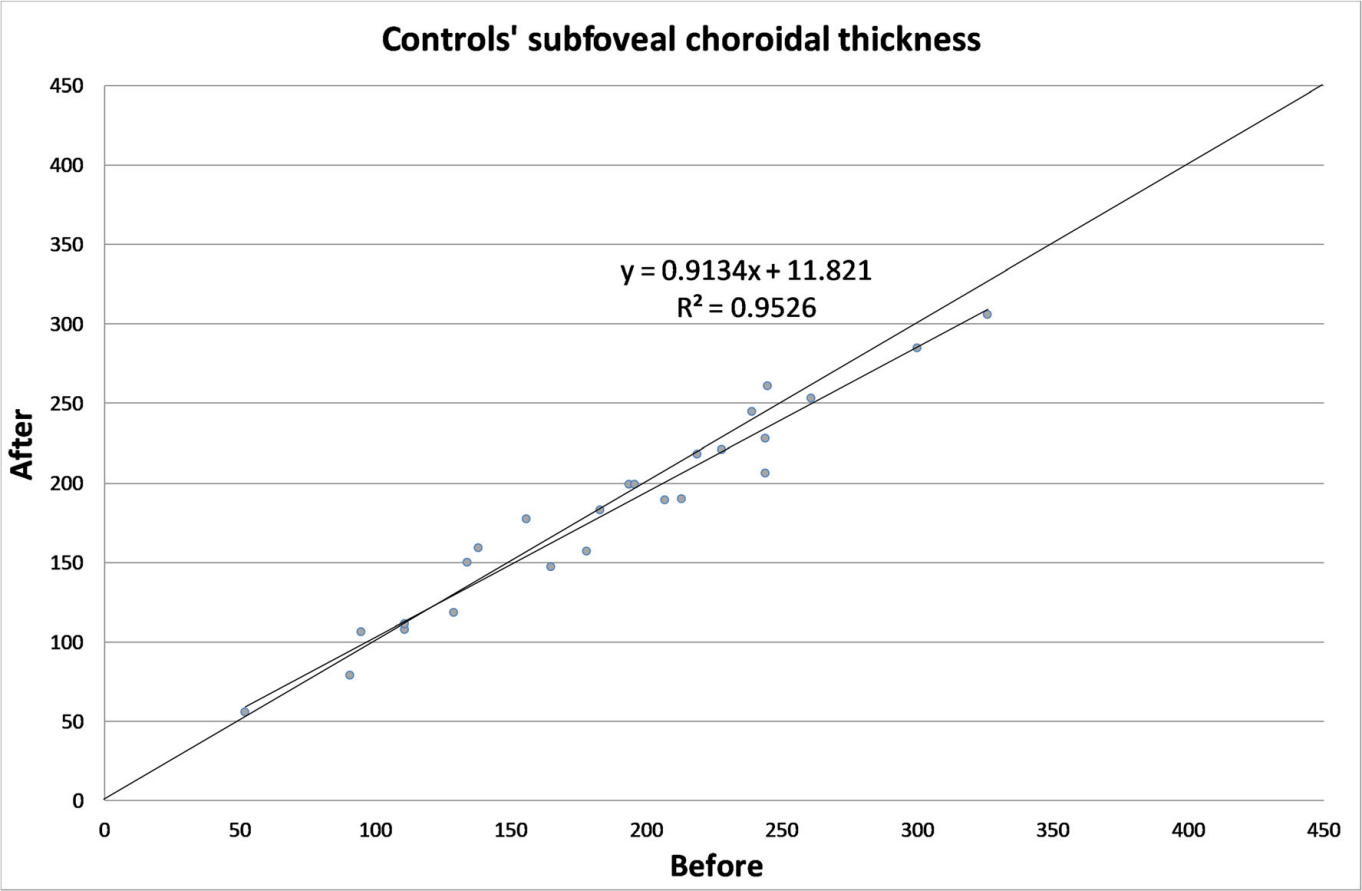
Control subject choroidal thickness at the subfoveal level at the first visit and 1 month later

**Fig. 6:**
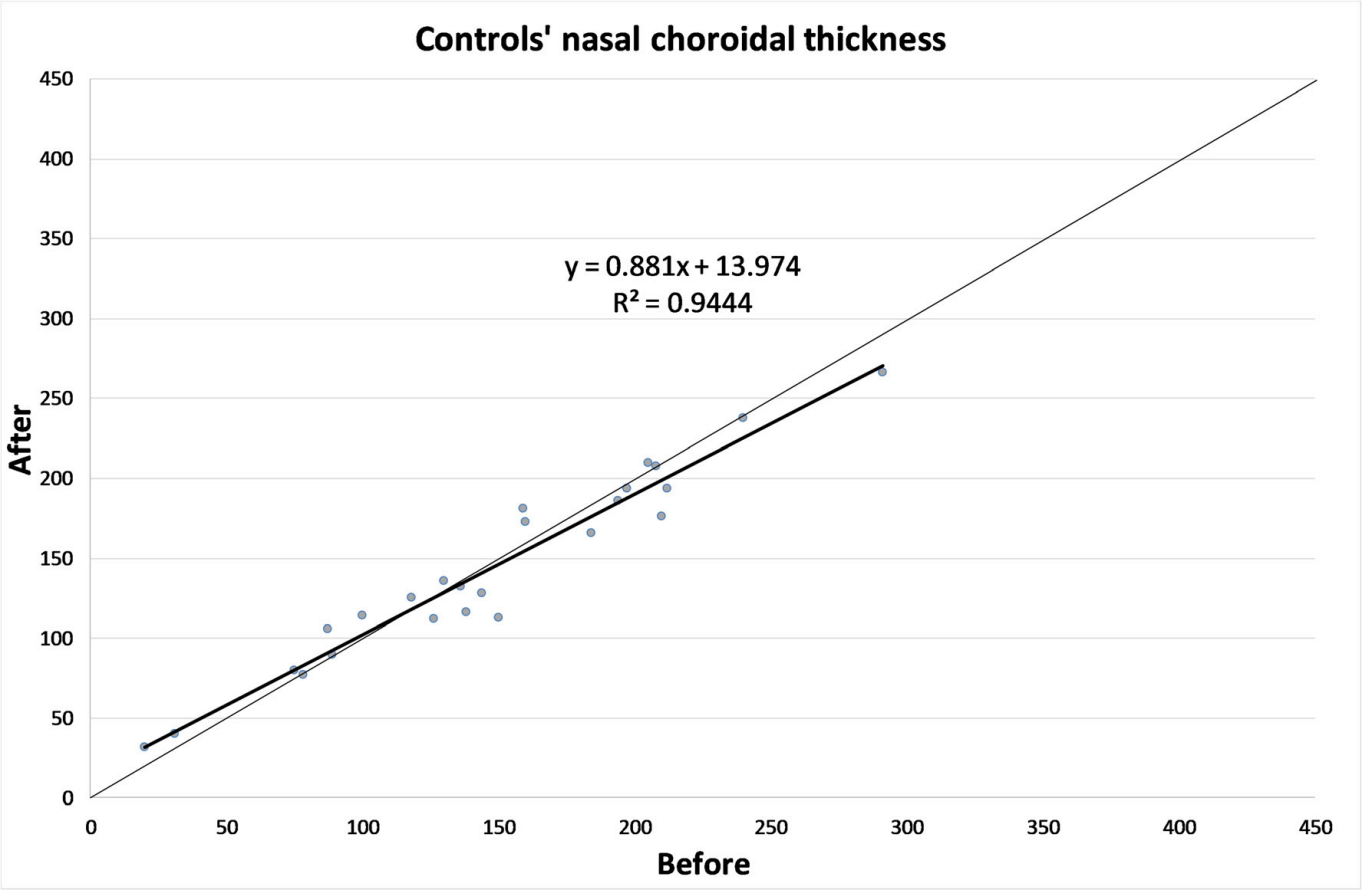
Control subject choroidal thickness at 1.5 mm nasally from the fovea at the first visit and 1 month later

**Fig. 7:**
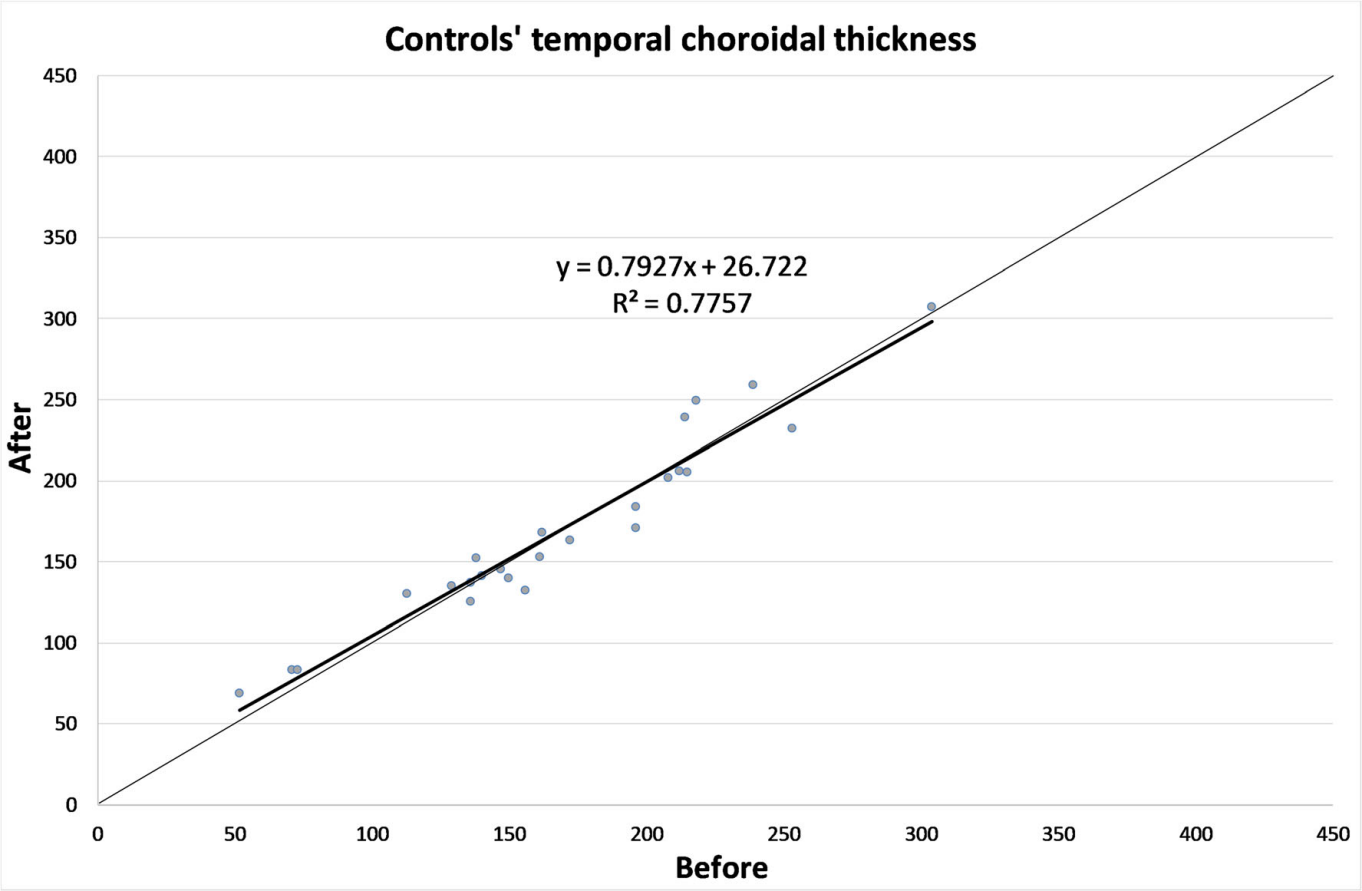
Control subject choroidal thickness at 1.5 mm temporally from the fovea at the first visit and 1 month later

**Fig. 8:**
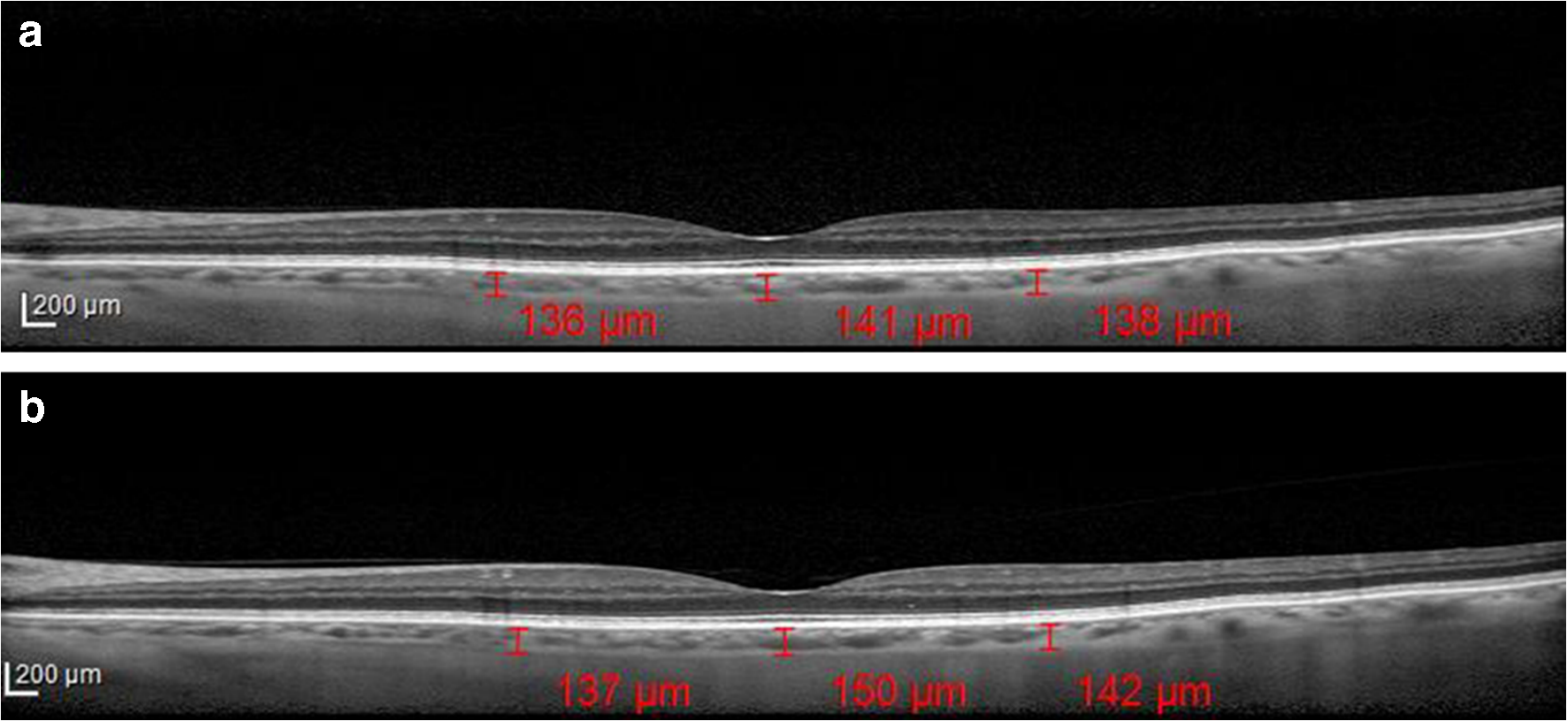
(**a**) Choroidal thickness evaluated at the subfoveal level, at 1.5 mm nasally and temporally from the fovea in a control subject at the first visit. **b** Choroidal thickness evaluated at the subfoveal level, at 1.5 mm nasally and temporally from the fovea in the same subject, 1 month later

The CT in different zones before and 1 month after the alpha-lytic therapy withdrawal is shown in Table 1 and in Figs. 1, 2, 3, 4a, b. The same values in the control group are shown in Table 2 and in Figs. 5, 6, 7, 8a, b. The data, analyzed with the Wilcoxon signed rank test, underlined a statistically significant CT reduction 1 month after therapy withdrawal (*p* = 0.05). No significant changes in the control group have been detected (Table 2).

**Table 1:** Choroidal thickness characteristics of patients before and after therapy withdrawal

		Preoperative visit [μm]	After therapy withdrawal [μm]	*p*
Fovea	Mean	224.1	217	0.025
	SD	79.7	80.4	
	Median	229	219	
	Range	62–387	53–392	
Nasal	Mean	198.2	193.8	0.039
	SD	83.8	82.8	
	Median	185	186	
	Range	62–375	74–357	
Temporal	Mean	217.1	204.7	0.003
	SD	55.9	54.4	
	Median	210	208	
	Range	90–312	58–301	

Mean, standard deviation (SD), median and range of choroidal thickness evaluated at the fovea, at 1.5 mm nasally and 1.5 mm temporally from the fovea at the preoperative visit and 1 month after alpha-lytic therapy withdrawal

**Table 2:** Choroidal thickness characteristics of control group.

		Preoperative visit [μm]	One month later [μm]	*p*
Fovea	Mean	187.3	182.1	0.113
	SD	68.8	63.8	
	Median	194	189	
	Range	52–326	56–306	
Nasal	Mean	148.1	146.0	0.647
	SD	65.2	58.8	
	Median	144	136	
	Range	20–291	32–266	
Temporal	Mean	168.2	169.8	0.667
	SD	59.5	58.5	
	Median	161	153	
	Range	52–304	69–307	

Mean, standard deviation (SD), median and range of choroidal thickness evaluated at the fovea, at 1.5 mm nasally and 1.5 mm temporally from the fovea at the pre-operative visit and 1 month later in the control group

Due to the small number of patients, and the absence of statistically significant difference in the AL between the two groups, evaluated with Mann-Whitney test (Table 3), they were not divided in subgroups according to AL or refractive errors.

**Table 3:** Axial length characteristics of the two groups

	Patients under alpha-lytic therapy [mm]	Control group [mm]
Mean	23.51	23.52
SD	0.92	1.51
Median	23.52	23.15
Range	21.17–25.41	21.72–28.76
P	0.294	

Mean, standard deviation (SD), median and range of the axial length of patients under alpha-lytic therapy and control group

## Discussion

Alpha-lytic therapy is a widely used medication for BPH treatment that has been shown to cause IFIS, which involves the anterior uveal portion. In rabbit eyes, choroidal vasodilation and reduced vascular tone related to α_1_ adrenoreceptor use were detected [7]. In two case reports a choroidal detachment in patients assuming Tamsulosin was described [8, 9].

The EDI-OCT, introduced in 2008, allows precise in vivo CT measurements and represents the most used method for choroidal imaging evaluation. It does not require SD-OCT hardware changes [10], because the software shifts the zero-delay line below, rather than above, obtaining inverted images. In this way, the operator can capture EDI-OCT images more easily because the image is now direct, rather than inverted. However, this technique does not allow the automatic CT measurement, so the operators have to manually draw the line to measure it [11]. In clinical practice, CT detection is the most useful application for EDI-OCT choroidal imaging. Two studies on CT changes alpha-lytic therapy related showed different results [12, 13].

Sari at al. [12] utilizing an EDI-OCT (ZEISS Cirrus HD-OCT 4000) in 29 eyes of 29 patients with new BPH diagnosis before and 3 months after tamsulosin intake attempted to check any drug-induced choroidal change. CT at the subfoveal level and at 750 μm nasally and temporally from the fovea was measured. All 3 observed points showed a significant thickness increase, and a vasomotor effect was suggested. In a comment to this paper, Yolcu et al. [14] proposed that tamsulosin affinity to melanin leads to its choroidal sequestration and its α1A adrenoreceptor-blocking activity leads to CT increase.

Dogan et al. [13] in 2017 evaluated 63 right eyes of 63 patients diagnosed with BPH (32 taking alfuzosin and 31 tamsulosin) with EDI-OCT (ZEISS Cirrus HD-OCT 4000). The measurements were performed manually, using a dedicated software, at the subfoveal level and at 3 mm nasally and temporally from the fovea. Contrary to the previous one, a statistically significant choroidal thickening was only observed in patients treated with Alfuzosin.

The differences between these two papers could be related to the measurement points, in one case 750 μm and in the other one, 3 mm from the fovea.

This hypothesis seems to be supported by the statistically significant CT reduction at 1.5 mm nasally and temporally from the fovea after drug withdrawal, found in the present study.

However, in our opinion, the main importance of the present study is the finding of a CT decrease when alpha-lytic therapy was discontinued.

In this study CT was not related to refractive errors of the examined patients. At the first sight, this could be seen as a limitation, but there are mainly two reasons for this choice. First of all, some lens opacities were present also in the fellow eye, but, more importantly, we decided to analyze the AL instead of refractive error because previous studies showed that CT presents a significant relationship with AL, whereas borderline significance was seen with the refractive errors [15–17].

The CT evaluation might be the key for the surgery timing in patients with IFIS risk. A smaller CT reduction might be associated with alpha-lytic therapy long lasting effects on the whole uvea, hence the surgeons could delay procedures until no residual drug effects are present, making the CT measurements an important step for this challenging problem resolution. In the light of our findings, to establish the landmark that could predict the IFIS absence during surgery, further studies comparing the CT decrease and the IFIS presence during surgery are needed.

## Data Availability

The data used to support the findings of this study are available from the corresponding author upon request.
